# Gain of function in *Mycobacterium bovis* BCG Moreau due to loss of a transcriptional repressor

**DOI:** 10.1590/0074-02760180267

**Published:** 2018-10-11

**Authors:** Renata Monteiro-Maia, Paloma Rezende Correa, Periela da Silva Sousa-Vasconcelos, Rosa Teixeira de Pinho, Leila Mendonça-Lima

**Affiliations:** 1 Fundação Oswaldo Cruz-Fiocruz, Instituto Oswaldo Cruz, Laboratório de Genômica Funcional e Bioinformática, Rio de Janeiro, RJ, Brasil; 2 Fundação Oswaldo Cruz-Fiocruz, Instituto Oswaldo Cruz, Laboratório de Imunologia Clínica, Rio de Janeiro, RJ, Brasil

**Keywords:** BCG Moreau, rv3405c, RV3406, THP-1 cells, tuberculosis

## Abstract

The Bacille Calmette-Guérin (BCG) vaccine comprises a family of genetically different strains derived by the loss of genomic regions (RDs) and other mutations. In BCG Moreau, loss of RD16 inactivates *rv3405c* ^*^ , encoding a transcriptional repressor that negatively regulates the expression of Rv3406, an alkyl sulfatase. To evaluate the impact of this loss on the BCG and host cell viability and the cytokine profile, THP-1 cells were infected with BCG Moreau (harbouring the empty vector) and a complemented strain carrying a functional copy of *rv3405c*. Viability of the host cells and bacteria as well as the pattern of cytokine secretion were evaluated. Our results show that the viability of BCG Moreau is higher than that of the complemented strain in an axenic medium, suggesting a possible functional gain associated with the constitutive expression of Rv3406. Viability of the host cells did not vary significantly between recombinant strains, but differences in the profiles of the cytokine secretion (IL-1β and IL-6) were observed. Our results suggest an example of a functional gain due to gene loss contributing to the elucidation of the impact of RD16 on the physiology of BCG Moreau.

Tuberculosis (TB) is caused by the intracellular pathogen *Mycobacterium tuberculosis* (Mtb) and remains a major public health problem. According to World Health Organization (WHO), 6.3 million new cases of TB were reported in 2016,^1^ and in the last two decades, the disease was responsible for more deaths than smallpox, malaria, plague, influenza and AIDS together.^2^ Brazil remains among the high burden countries with an incidence of 42/100,000 inhabitants in 2016.^3^

Bacille Calmette-Guérin (BCG) is the only available vaccine against TB and the most widely used vaccine worldwide.^4^ Modern BCG comprises a group of different vaccine strains;^5^ ^,^ ^6^ all strains are derived from an original attenuated “BCG” obtained after sequential *in vitro* passages of a virulent *Mycobacterium bovis* isolate in a medium containing ox bile.^7^ The attenuated strain was successfully used in humans for the first time in 1921 and, after 1925, was distributed to various laboratories worldwide and maintained under different culture conditions.^8^ ^,^ ^9^. By the time standardised seed-lot production methods were implemented in the 1960s, BCG sub-strains had accumulated a variety of specific genetic differences that partially explain the variable efficacy of the vaccine.^10^

BCG Moreau, the Brazilian vaccine strain,^11^ has a specific genomic deletion (RD16) comprising 7,608 bp^6^ resulting in the fusion of *rv3400*-*rv3405c* and loss of the intervening genes.^12^ Previous studies by our group have established that Rv3405c is a transcriptional regulator that represses the expression of the adjacent *rv3406* gene.^13^ As a consequence, BCG Moreau accumulates Rv3406 due to its constitutive expression; in comparison, BCG Pasteur does not produce Rv3406 under the standard culture conditions. Complementation of BCG Moreau with a functional copy of *rv3405c* obtained from BCG Pasteur restores the regulation and abolishes the Rv3406 accumulation.^13^

The *rv3406* sequence encodes a protein identified as an alkyl sulfatase.^14^ Sulfatases catalyse the hydrolysis of various molecules ranging from small cytosolic steroids (oestrogen sulfate) to complex cell-surface carbohydrates (glycosaminoglycans - GAG). These substrates contain a wide variety of sulfate esters including hydrophobic glucosinolate, steroid and thyronine sulfates, amphiphilic sulfated carbohydrates found in GAGs, proteoglycans, glycolipids and water-soluble mono- and disaccharide sulfates.^15^

The aim of the present study is to evaluate the functional impact of the loss of Rv3405c on the *M. bovis* BCG Moreau viability and its initial interaction with the host cells using a THP-1 cell model, monitoring the behaviour of the bacilli and the host cells in response to infection. Two BCG Moreau recombinant strains^13^ were compared in this study: the first strain (M::*05c*) harbours a functional copy of *rv3405c* from the BCG Pasteur cloned in the pUS972 plasmid,^16^ and the second strain carries the empty vector (MD*05c*). Both strains were grown in the 7H9 medium supplemented with 10% ADC and 0.05% Tween-80 in the presence of 25 μg mL^-1^ kanamycin. For growth curves, three aliquots of each strain were defrosted and maintained independently for two weeks of culture expansion. Bacteria concentration was adjusted to an optical density (OD_600nm_) of 0.2, and culture volumes were expanded to 60 mL of 7H9 medium. OD was evaluated every 24 h for two weeks.

Cells of a human myelomonocytic cell line, THP-1, were grown as suspension cultures in RPMI medium supplemented with 10% heat-inactivated foetal bovine serum (FBS) (Gibco), 100 μg mL^-1^ penicillin and 100 μg mL^-1^ streptomycin (Sigma), 25 mM Hepes (Sigma) and 2 mmMol L^-1^ glutamine (Sigma) at 37ºC in a humidified atmosphere of 5% CO_2_. THP-1 cells were differentiated into macrophages using 200 nM of phorbol 12-myristate 13-acetate (PMA; Sigma) for 48 h followed by a 48 h incubation in the medium without PMA (protocol adapted from Daigneault et al.^17^). Cells were infected at a multiplicity of infection (moi) of 1/1 with each BCG strain for 4 h and then washed thrice with RPMI to remove non-internalised bacteria. No differences in internalisation rates were observed between the recombinant strains [Supplementary data (Fig. 1)]. At specific time points, samples were collected for bacterial and cell viability determination and cytokine assays.

Bacterial intracellular viability was evaluated at 4, 6, 24, 48, 72 and 96 h after the infection of 2 x 10^5^ THP-1 cells. At these time points, cells were lysed with 0.05% SDS and bacteria were recovered after centrifugation for 10 min at 16.000 x g. For CFU determination, bacteria in the pellet fraction were serially diluted and plated on the 7H10 medium supplemented with 10% ADC and 25 μg mL^-1^ kanamycin; the colonies were counted after 28-30 days of incubation at 37ºC.

To evaluate macrophage viability during the course of infection, 3.2 x 10^4^ cells were plated onto black 96-well culture plates. At 24, 48, 72 and 96 h after the infection, 100 µL of PrestoBlue diluted 1:10 (Life Technologies) was added to the infected cells for 1 h, and then viability was evaluated with a FlexStation 3 microplate reader. Cells incubated with 0.01% Triton X-100 were used as zero viability controls; non-infected cells were used as a positive control and medium was used as a negative control.

**Fig. 1:**
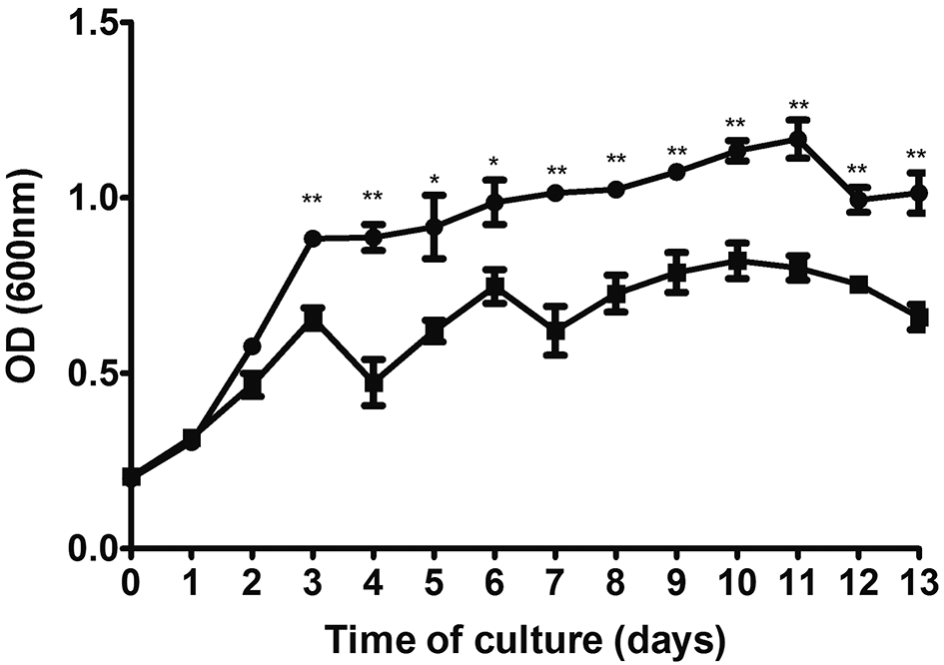
comparison of growth rate of recombinant Bacille Calmette-Guérin (BCG) strains MD*05c* and M::*05c* in axenic medium for two weeks. Circles (●) indicate the MD*05c* strain, and squares (■) indicate the M::*05c* strain. Data represent the average of three biological replicates +/- SD. T-Test = *p < 0.05; **p < 0.01.

To evaluate cytokine secretion, 3.2 x 10^4^ cells were plated onto 96-well culture plates and infected with each recombinant BCG strain as described above; at specific time points (6, 24 and 72 h), supernatants were removed, centrifuged for 2 min at 700 x g at 4ºC and stored at -70ºC for subsequent cytokine and chemokine analyses. The production of the cytokines (IL-1β, IL-6, IL-10 and TNF-α) and of the chemokine MIP-1β was measured by enzyme-linked immunosorbent assay (ELISA) using a duo-set kit from R&D Systems (cat nos: IL-1β, DY 201; IL-6, DY 206; IL-10, DY 217B; TNF-α, DY210; MIP-1β, DY 271) according to the manufacturer recommendations. The limits of the detection of the cytokines were as indicated by the manufacturer: IL-1b = 3.91 - 250 pg mL^-1^; IL-6 = 9.38-600 pg mL^-1^; IL-10 = 31.2-2000 pg mL^-1^; TNF-α = 15.6-1000 pg mL^-1^; and MIP-1b = 15.6-1000 pg mL^-1^. Spectrophotometer (Spectra Max 190) readings were taken at 450 nm.

Statistical differences were calculated by T-test or two-way ANOVA and Bonferroni post-test (as specified in figure legends) using the Graph Pad Prism 5.0 Software (San Diego, CA, USA). Graphs represent at least three independent experiments. Data were considered significant if p < 0.05.

Comparison of the growth profiles of two BCG strains in an axenic medium shows a significant difference after three days and up to 13 days of culture, indicating a growth advantage for the strain MD*05c* that lacks the functional repressor (Fig. 1).

Ability to survive and multiply in an intracellular environment was assessed by infecting the THP-1 cells. At specific time points after the infection, the macrophages were lysed, bacteria recovered and CFUs determined by plating of serial dilutions as described above. The kinetics of viability indicates that, although MD*05c* lacks the functional repressor, it shows higher viability than M::*05c*; no statistically significant differences between the strains were observed (Fig. 2). Nevertheless, the data suggest that the presence of the functional Rv3405c transcriptional repressor impacts intracellular survival of BCG; however, this finding should be confirmed in additional experiments to become statistically significant.

**Fig. 2:**
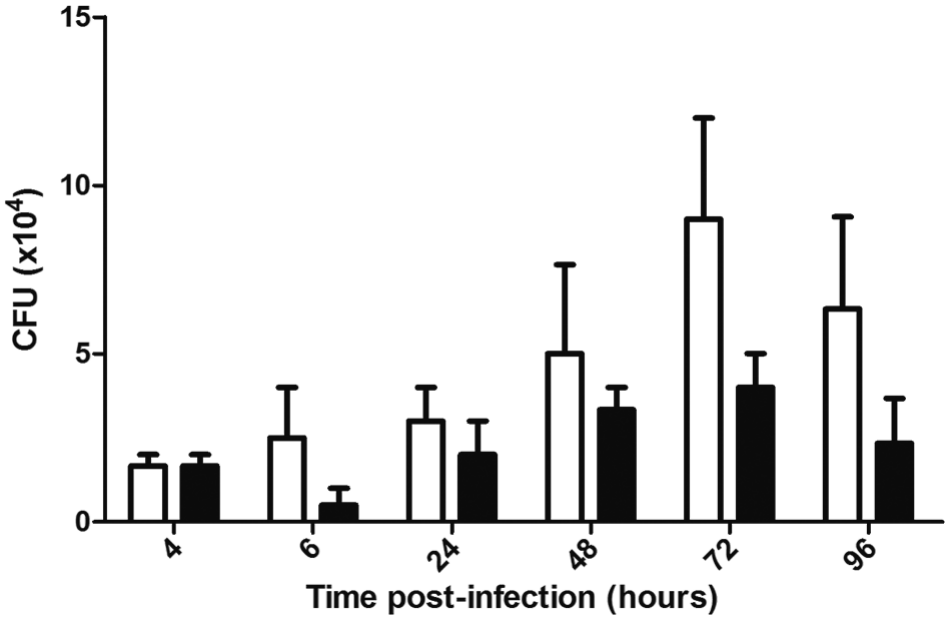
intracellular viability of recombinant Bacille Calmette-Guérin strains (BCGs) in the THP-1 cells. Macrophages were infected with recombinant BCGs for 4 hours, and bacterial viability was evaluated at specific time points post-infection. No significant differences were observed. White bars represent the MD*05c* strain, and black bars represent the M::*05c* strain. The results represent the average of three independent experiments +/- SD. Non-paired T-test.

Our results show a significant impact on macrophage viability due to the infection with BCG when compared to the non-infected cells, but no significant differences could be associated with specific BCG strain used (Fig. 3).

Cytokine responses were evaluated in the host cells infected with two recombinant BCGs and non-infected THP-1 cells. The levels of secreted IL-10, MIP-1β and TNF-α did not show any significant differences (data not shown). However, differences in the IL-1β and IL-6 production after 72 h of infection were observed. The BCG strain MD*05c* induced higher expression of IL-1β but lower expression of IL-6 compared to both the non-infected cells and the cells infected with the complemented strain (M::*05c*) (Fig. 4).

**Fig. 3:**
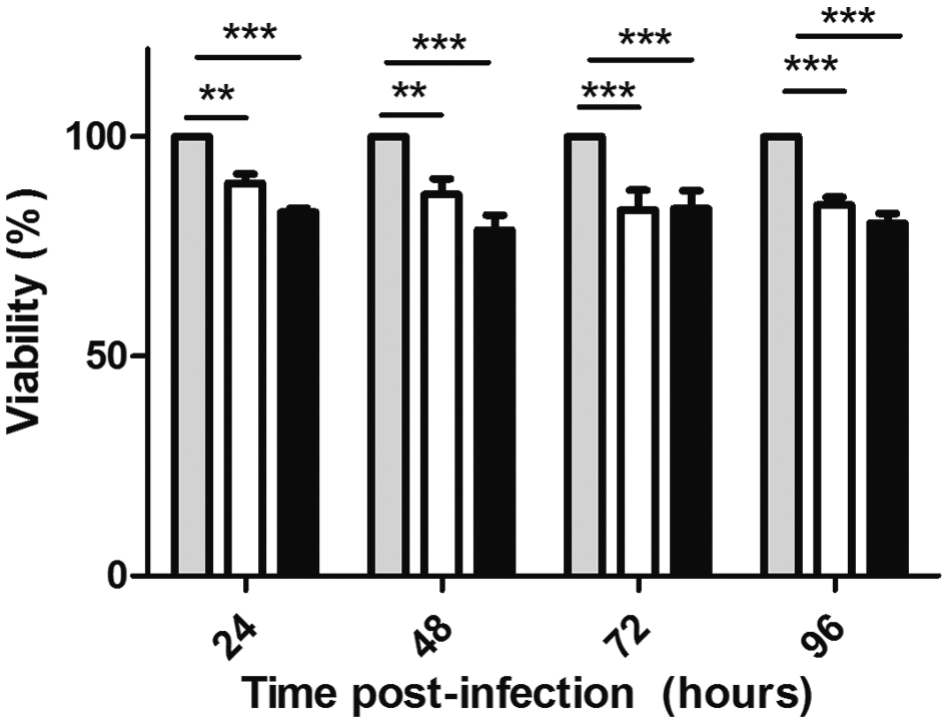
macrophage viability evaluated with PrestoBlue at 24, 48, 72 and 96 h post-infection. The data are viability of macrophages (%) infected with the Bacille Calmette-Guérin (BCG) strains MD*05c* (white bars) and M::*05c* (black bars) relative to non-infected controls (grey bars) and represent the mean (+/- SD) of four independent experiments. Two-way Anova +/- SD. Bonferroni post-test **p < 0.01; ***p < 0.001.

In an effort to further characterise the functional impact of mutations mapped in the genome of BCG Moreau, we evaluated two constructs that differ from each other only by the presence of a functional copy of *rv3405c*, a transcriptional repressor which regulates *rv3406* expression. Rv3406 was characterised as an alkyl sulfatase by Sogi et al.^14^ Sulfatases hydrolyse sulfate esters^18^ and have been implicated in the modification of the sulfate groups on glycosaminoglycans (GAGs), which are involved in developmental cell signalling and patterning phenomena in the extracellular matrix (ECM) and may play certain roles in bacterial pathogenesis.^15^ Additionally, sulfatases are involved in redox reactions and can be considered as alternative sources of sulfur important for biosynthesis of the essential amino acids and sulfur-containing cofactors.^19^

Our results indicate that the absence of the Rv3405c transcriptional repressor confers a growth advantage to the BCG Moreau in the axenic medium and in the intracellular environment of the THP-1 cells compared to the BCG Moreau complemented with a functional copy of *rv3405c* (M::*05c*). One explanation is that the absence of *rv3405c* and consequent constitutive expression of Rv3406 [Supplementary data (Fig. 2)] may contribute to enhanced mycobacterial adaptation possibly by conferring a metabolic advantage related to sulfur metabolism and/or carbon acquisition. This protein may be particularly important because it is the only annotated type II sulfatase in Mtb,^14^ even though mycobacterial genomes have an unusually high number of genes predicted to encode sulfotransferases and sulfatases.^19^ Moreover, recent drug screening studies have identified mutations that inactivate *rv3405c* in the sequenced Mtb strains which are resistant to new classes of compounds,^20^ ^,^ ^21^ implicating Rv3406 in the resistance mechanism. It is important to consider that other genes apart from *rv3406* may also be under the regulatory control of Rv3405c, and we are pursuing the studies aiming to map this possible regulatory network under the axenic and intracellular conditions.

**Fig. 4:**
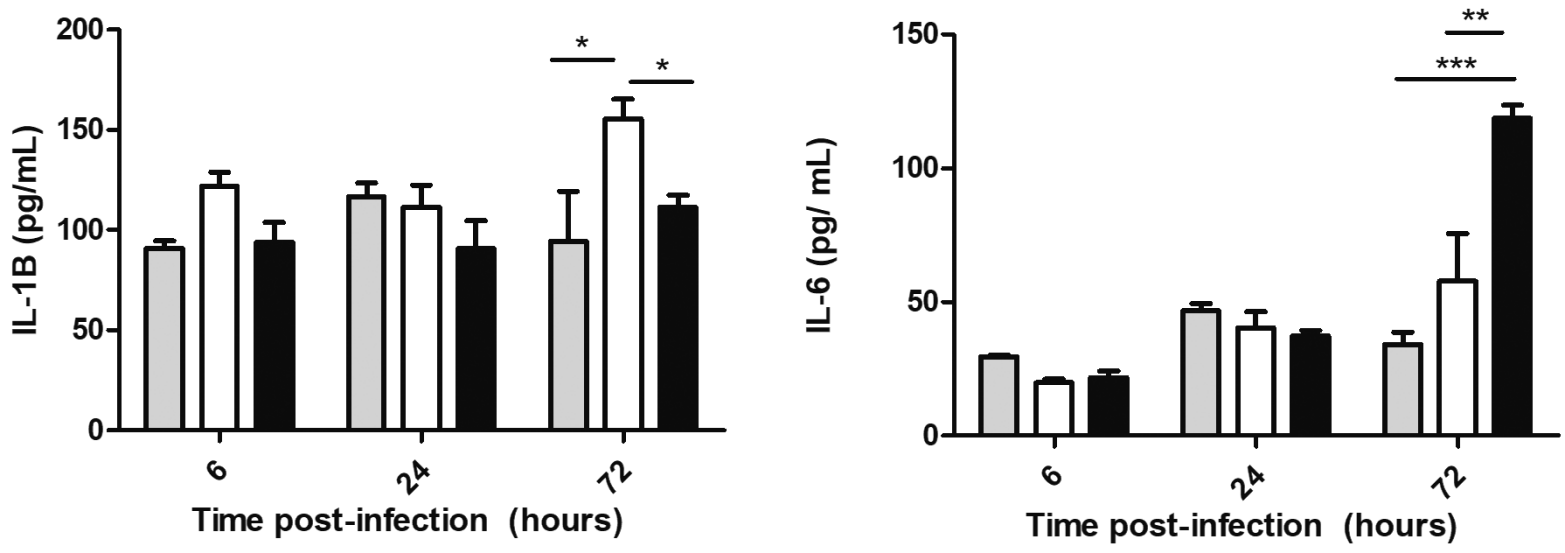
IL-1β (A) and IL-6 (B) were measured in the culture supernatants from the THP-1 macrophages that were not infected (grey bars) or infected with Bacille Calmette-Guérin (BCG) MD*05c* (white bars) and M::*05c* (black bars) at 6, 24 and 72 h post infection by enzyme-linked immunosorbent assay (ELISA). Bars represent the mean (+/- SD) of three independent experiments. Two-way Anova +/- SD. Bonferroni post-test *p < 0.05; **p < 0.01; ***p < 0.001.

Our approach has some limitations, including the use of macrophages derived from a cell lineage, THP-1. Nevertheless, our data provides an insight into the behaviour of BCG in response to the intracellular environment and the behaviour of the host cells in response to the bacterial infection. Protocols for the differentiation of the macrophages from the THP-1 cells are quite diverse in the literature, with variable PMA concentrations of 30 nM,^22^ 80 nM,^23^ 100 nM^24^ and 200 nM;^17^ additionally, the protocols differ in the duration of the exposure and post-differentiation incubation time. Lack of a uniform protocol has a strong impact on the interpretation of the results hindering comparison between various studies. Numerous studies have focused on macrophage activation and death in response to *M. tuberculosis*, but much less is known about the BCG-mediated cellular activities in the phagocytic cells.^25^ Under the experimental conditions used in our study, significant loss in macrophage viability was observed at all times due to the infection, but no significant loss in macrophage viability was observed due to the recombinant BCG strain used (Fig. 3). Other studies using BCG infected THP-1 cells report similar results.^26^ ^,^ ^27^.

It is well known that cell-mediated immune responses are involved in generating protection against Mtb with broad participation of the T_H_1 cells and pro-inflammatory cytokines such as IL-1β, IL-6, TNF-α and IFN-γ. These factors increase the microbicidal capacity of the macrophages through production of reactive oxygen and nitrogen intermediates that render the intracellular environment hostile^28^ and play a role in the formation of granulomas and subsequent containment of bacilli dissemination.^29^

In a previous study, Sousa-Vasconcelos et al.^22^ showed that BCG Moreau is capable of increasing the IL-1β and IL-6 levels in the supernatant of the THP-1 cells compared to the non-infected cells. IL-1β is a pro-inflammatory cytokine involved in the control of bacterial load through the recruitment of microbial agents.^30^ It is an important mediator of inflammation and plays a role in generating resistance to the Mtb infections. Our results showed an increase in this cytokine at 72 h post-infection possibly resulting in a decrease in bacterial viability observed at 96 h (p > 0.05). IL-6 is a pleiotropic cytokine performing a range of functions related to inflammation, host defence and tissue injury. Additionally, it plays an important role in the metabolism of carbohydrates and lipids by increasing lipolysis and the release of free fatty acids and glycerol.^31^ Our data show that IL-6 secretion is higher in the cells infected with the complemented strain M::*05c* than in the cells infected with BCG Moreau lacking a functional repressor. Since the presence of the functional repressor abolishes expression of the Rv3406 sulfatase, we could speculate that this loss is partially compensated by the increased expression of IL-6 through its effects on lipid metabolism and release of fatty acids.

Hayashi et al.^32^ compared cytokine secretion by the THP-1 cells in response to various BCG strains, reporting higher levels of IL-1β but lower levels of IL-6 secretion induced by BCG Moreau than by BCG Pasteur. Considering that BCG Pasteur carries a functional Rv3405c repressor, we obtained a similar cytokine profile. BCG Moreau and Pasteur differ in numerous genetic loci apart from r*v3405c,* making it almost impossible to establish the differences that are responsible for each observed phenotype. Our approach involves comparing two BCG Moreau constructs differing only in the presence of an active transcriptional repressor; it provides an opportunity to assess the impact of the loss of Rv3405c on some aspects of BCG Moreau physiology. Overall, our data indicate that constitutive expression of a type II sulfatase, Rv3406, favours bacterial viability and leads to a differential cytokine response in an *in vitro* model of infection. Increased viability may have a positive impact on vaccine efficacy. This is an example of functional gain due to gene loss that contributes to the detailed characterisation of the *M. bovis* BCG strain used in Brazil for production of vaccine against tuberculosis.
