# Supplementary material for: Gain of function in Mycobacterium bovis BCG Moreau due to loss of a transcriptional repressor
Source: Mem Inst Oswaldo Cruz. 2018 Oct 11;113(11):e180267. doi: 10.1590/0074-02760180267 (PMC6180650; doi:10.1590/0074-02760180267)
Supplement: Supplementary file 1 [file 1678-8060-mioc-113-11-e180267-s.pdf]

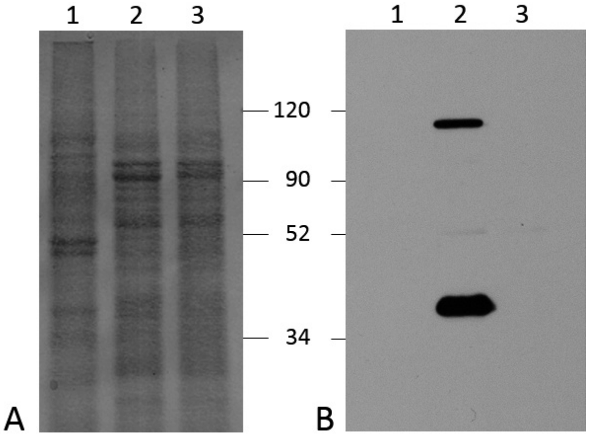

Fig. 1: internalisation rates of the recombinant strains MD*05c* and M::*05c*. Internalisation rate was calculated by dividing the number of bacteria used in the infection by the number of bacteria recovered at 4 hours post-infection after removal of non-internalised bacteria. No differences in internalisation rates were observed between the recombinant strains. Statistical analysis: Unpaired T-test. +/- standard deviation (DP).

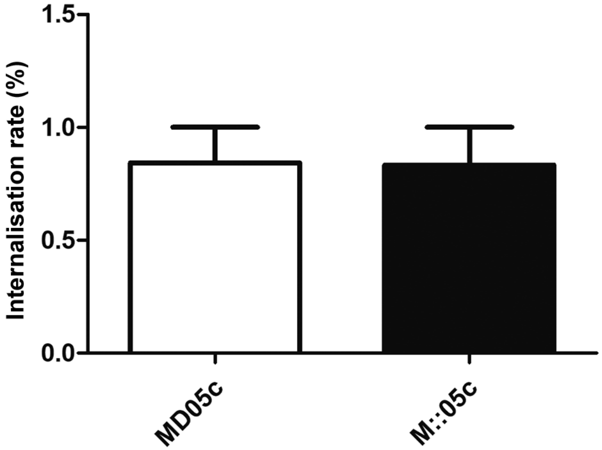

Fig. 2: Rv3406 protein expression. Macrophages were infected with the recombinant Bacille Calmette-Guérin (BCG) strains MD*05c* (lane 2) and M::*05c* (lane 3). Non-infected cells were included as a control (lane 1). After 96 h, cells were lysed, 50 µg of total protein separated by sodium dodecyl sulfate polyacrylamide gel electrophoresis (SDS-PAGE) (15%), transferred to a nitrocellulose membrane and stained with MemCode (A). Western blotting was done using a mouse polyclonal anti-Rv3406 antibody (B). MW standards are indicated in kDa.

CC BY
